# Sub-millisecond Control of Neuronal Firing by Organic Light-Emitting Diodes

**DOI:** 10.3389/fbioe.2019.00278

**Published:** 2019-10-22

**Authors:** Bruno F. E. Matarèse, Paul L. C. Feyen, John C. de Mello, Fabio Benfenati

**Affiliations:** ^1^Department of Chemistry, Imperial College London, South Kensington Campus, London, United Kingdom; ^2^Center for Synaptic Neuroscience and Technology, Istituto Italiano di Tecnologia, Genoa, Italy; ^3^Section of Physiology, Department of Experimental Medicine, University of Genova, Genoa, Italy; ^4^Centre for Organic Electronic Materials, Department of Chemistry, Trondheim, Norway; ^5^IRCCS Ospedale Policlinico San Martino, Genoa, Italy

**Keywords:** organic light-emitting diodes, optogenetics, neurons, pulsed-operation, optical stimulation, bioelectronics, photoexcitation, electrophysiology

## Abstract

Optogenetics combines optics and genetics to enable minimally invasive cell-type-specific stimulation in living tissue. For the purposes of bio-implantation, there is a need to develop soft, flexible, transparent and highly biocompatible light sources. Organic semiconducting materials have key advantages over their inorganic counterparts, including low Young's moduli, high strain resistances, and wide color tunability. However, until now it has been unclear whether organic light emitting diodes (OLEDs) are capable of providing sufficient optical power for successful neuronal stimulation, while still remaining within a biologically acceptable temperature range. Here we investigate the use of blue polyfluorene- and orange poly(p-phenylenevinylene)-based OLEDs as stimuli for blue-light-activated Sustained Step Function Opsin (SFFO) and red-light-activated ChrimsonR opsin, respectively. We show that, when biased using high frequency (multi-kHz) drive schemes, the OLEDs permit safe and controlled photostimulation of opsin-expressing neurons and were able to control neuronal firing with high temporal-resolution at operating temperatures lower than previously demonstrated.

**Graphical Abstract:**
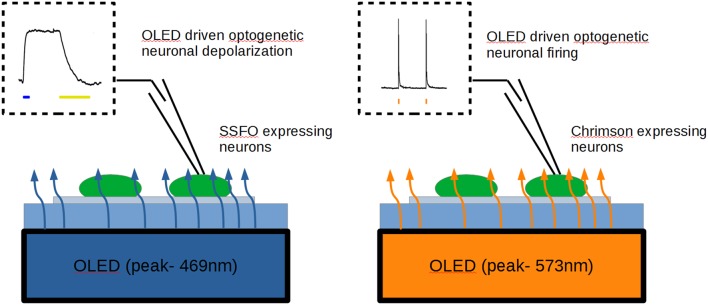
Using blue polyfluorene- **(left)** and orange poly(p-phenylenevinylene) **(right)** OLEDs in pulsed-mode operation, we obtained a successful activation of Sustained Step Function Opsin (SFFO) and ChrimsonR opsin, respectively, yielding depolarization and millisecond control of firing in opsin-expressing neurons by OLEDs.

## Highlights

- Successful activation of Sustained Step Function Opsin (SFFO) by blue polyfluorene-OLEDs.
- Successful activation of ChrimsonR opsin by orange poly(p-phenylenevinylene)-OLEDs.
- Pulsed-mode operation allows neuronal stimulation in a compatible temperature range.
- Safe and controlled millisecond control of firing in opsin-expressing neurons by OLEDs.

## Introduction

Optogenetics involves the genetic modification of a cell to enable optical control of ion currents across the plasma membrane by light-gated ion channels or transporters. The applications of optogenetics are diverse and include the ability to investigate neural circuits with high spatiotemporal control, the treatment of a wide range of degenerative and paroxysmal pathologies, the regulation of artificial organ function, or the triggering of bio-hybrid robot movements (Toettcher et al., 2011; Reinbothe et al., 2014; Cehajic-Kapetanovic et al., 2015; Paz and Huguenard, 2015; Park et al., 2016). The power of optogenetics derives in part from its ability to genetically target specific cell types and also from the availability of a broad range of protein tools that have been engineered to tune kinetics, ion selectivity, optical response, and wavelength sensitivity (Mattis et al., 2011; Yizhar et al., 2011; Klapoetke et al., 2014; Gaub et al., 2015). With the technology already being used in a clinical testing for the treatment of degenerative blindness (see RST-001 Phase I/II Trial for Retinitis Pigmentosa, 2016), there is a recognized need to develop thin, flexible, efficient, multi-color biocompatible light sources for both wearable and implantable devices (Park et al., 2009; Someya et al., 2016).

Spectrally matched stimulation of photosensitive proteins is typically carried out with filtered halogen lamps, lasers or inorganic light emitting diodes, with the light beam commonly delivered via microscopes or by coupling to optical fibers (Dal Maschio et al., 2010; Welkenhuysen et al., 2016). There have also been several reports involving the use of arrays of micro LEDs to target individual neurons with high spatial precision (Grossman et al., 2010; Gossler et al., 2014; Soltan et al., 2014; Qazi et al., 2018). While the initial results were promising, various drawbacks including the bulky and rigid features of inorganic LED sources, heat production, decrease in efficiency or mismatched spectral characteristics (Krames et al., 2007; Tsintzos et al., 2008; Fan and Li, 2015) have so far prevented the development of fully implantable devices.

By contrast, organic light-emitting diodes (OLEDs) can be fabricated using layers of soft conformable materials that are well-matched to the prerequisites of implantable devices. The thin layers used in the OLED device stack add minimal (sub-micron) thickness to the chosen substrate, making it feasible to incorporate OLEDs in many locations where larger light-sources could not be tolerated (Yang et al., 2012; Zhang et al., 2015). Moreover OLEDs can be processed onto a wide range of substrate materials, allowing for the fabrication of flexible (Yokota et al., 2016) and stretchable devices (Someya et al., 2016; Yin et al., 2016). Using standard processing methods, it is possible to fabricate homogeneously emitting, pixelated surfaces covering microscopic (μm^2^) to macroscopic (cm^2^) length scales (Sandström et al., 2012; Zheng et al., 2013), with overall device thicknesses as low as 2 μm (White et al., 2013). From a biological perspective, this favorably positions the technology for many useful tasks, ranging from highly localized sub-cellular targeting to organ targeting (Toettcher et al., 2011; Bruegmann et al., 2015). Rapid progress has been made in applying organic electronics to optogenetic activation in systems including green algae, *Drosophila* larvae and Human Embryonic Kidney-293T (HEK293T) cells (Steude et al., 2015, 2016; Morton et al., 2016) but, until now, the chosen optical stimuli have lasted seconds to minutes, limiting the range of optogenetic applications (Smith et al., 2014; Blain Christen et al., 2016). The performances of OLEDs are progressing rapidly and have now reached the market enabling new display and lighting application (e.g., smartphone display and large-size OLED TVs), but their operating optical power and temporal resolution for optogenetics requirements are insufficiently investigated. In particular, the feasibility of using sub-millisecond frequency biasing schemes to improve electroluminescence efficiencies, and hence permit short-timescale stimulation of opsins, has not previously been investigated.

One key challenge toward the long-term goal of developing an implantable OLED-based optical stimulus for controlling neural activity is to identify simple and biocompatible OLED structures that are capable of eliciting biological effects. Here, we report, for the first time, the use of poly(p-phenylene vinylene)-based and polyfluorene-based light emitting polymers for generating polymeric OLEDs for opsin stimulation. New possibilities for organ targeting stimulation are offered by homogeneous solution-processed large-area polymeric OLED pixels that are difficult to achieve by non-solution processed OLEDs or their inorganic LED counterparts. Moreover, high elastic modulus and biological inertness of carbon-based polymers confer major advantages for use in non-conformal body cavities.

Using extended multi-kHz pulse trains, we demonstrate an effective control of the firing rate of primary neurons expressing microbial opsins *in vitro* on a millisecond time scale.

To test our OLEDs, two opsins were selected from the huge toolbox of opsin variants characterized by distinct spectral sensitivity and different kinetics, namely the blue-sensitive, bi-stable Sustained Step-Function Opsin (SSFO; Yizhar et al., 2011) to induce multi-second neuronal depolarization with high sensitivity, and the red-sensitive, fast kinetics ChrimsonR (Klapoetke et al., 2014) to induce a sub-millisecond control of neuronal firing. The results reported here confirm the feasibility of using OLEDs operating at low, biologically compatible temperatures to control neural activity.

## Materials and Methods

### Materials

Materials employed: MEH-PPV (poly[2-methoxy-5-(2-ethylhexyloxy)-1,4-phenylenevinylene]) for F(4); CN-PPV [poly(2,5-di(hexyloxy)cyanoterephthalylidene)] for F(5); PFO [poly(9,9-di-n-octylfluorenyl-2,7-diyl)]; and PVK [poly(9-vinylcarbazole)] for F(1) were ordered from Sigma Aldrich. Super Orange (SO): Phenyl alkoxyphenyl PPV copolymer, conjugated polymers Livilux™ PDO-124 (SO-PPV) for F(2) devices were obtained from Merck PPF. For b**i**ocompatibility assays, thin films of fluorescent polymers (PFO, PVK, SO-PPV) were prepared by dissolving 6 mg material in 1 mL Chlorobenzene. Glass coverslips were spin-coated for 30 s at 1,200 RPM, and annealed at 150°C for 20 min.

### OLED Fabrication

Conventional solution processing organic light emitting diodes were fabricated on glass by coating indium tin oxide (ITO; 10 ohm/sq) with a 30 nm layer of poly(3,4-ethylendioxythiophene:poly(styrenesulfonate) (PEDOT:PSS). All spin coating steps were carried out in air. Cleaning of the ITO slides; film coating and electrode deposition were carried out all the same day to ensure optimum results. ITO coated glass substrates were supplied by Xin Yan Technology Ltd. The glasses were sonicated in standard acetone and then in soap and double-distilled (DI) water for 20 min, in DI water 3 times for 5 min, in acetone twice for 5 min and in isopropanol (IPA) for 5 min, followed by blow-drying of the substrates. The ITO substrates were treated by oxygen plasma treatment. The PEDOT:PSS layers were deposited by spin coating at a spin speed of 3,500 rpm for 40 s and then annealed at 150°C for 20 min. Polymeric devices followed conventional PLED structure using PEDOT:PSS for the injection of holes from the ITO anode and Calcium for the injection of electron from the Aluminum cathode. The precursor solutions were prepared the day before device fabrication and the solutions were stirred at <50°C overnight. The precursor solutions of fluorescent polymers (PFO, PVK, SO-PPV, MEH-PPV, and CN-PPV) were prepared by dissolving 6 mg material in 1 mL chlorobenzene and were spin-coated for 30 s at 1,200 RPM on top of PEDOT:PSS. Following active layers deposition, Calcium (25 nm) and aluminum (100 nm) top-electrodes were deposited onto all the devices by thermal evaporation through a shadow mask at 10-6 mbar. Active areas measured 75 mm^2^. The encapsulation of the devices was effected manually using another glass substrate of 1.1 mm adhered onto the device using flexible sealants from DELO-KATIOBOND and a low power UV light to set the glue.

### OLED Characterization

The electrical characteristics of the devices encapsulated were measured in air at room temperature using a Keithley 2450 electrometer for the electrical current-voltage measurement. Emission spectra were measured using a fiber-coupled Ocean Optics 2000+ spectrometer. Absolute optical power intensity was determined using a large area calibrated Si PIN Photodiode (28 × 28 mm active area, from Hamamatsu) coupled to a Keithley 6517A electrometer. An OPT101P Photodetector Amplifier from Texas Instruments connected to the National Instruments NI PCI-5112 100MHz Digital Oscilloscope Board was used to detect fast pulsing mode imposed by the Arduino Uno microcontroller. Surface temperature was measured and digitized by placing a thermistor (TC-3444B, Warner Instruments) in contact with the OLED anode-side glass surface. Output voltage trace responses were analyzed in Matlab (Mathworks). For recordings in culture medium, 500 μl of Hank's Balanced Salt Solution (HBSS) was used to immerse the sensor.

### Primary Neuronal Cultures

Polymeric films and glass culture substrates were thermally sterilized at 120°C, prior to overnight incubation in 0.1% poly-L-lysine solution. Primary cultures of hippocampal neurons were prepared from embryonic 18-day rat and mouse embryos (Charles River). Briefly, hippocampi or cortex were dissociated by a 15-min incubation with 0.25% trypsin at 37°C and cells were plated on poly-L-lysine-coated substrates (0.1% PLL in Borax solution overnight) in Neurobasal supplemented with 2 mM L-glutamine, 2% B27, 100 μg/ml penicillin and 100 μg/ml streptomycin, and with 10% horse serum (Life Technologies) in the first 4 h of plating. Cultures were maintained at 37°C in a humidified atmosphere containing 5% CO_2_.

### Expression of Opsins in Neurons

Neurons were transfected by Lipofectamine 2000 (Thermo Fischer Scientific) reagent using a 1 h incubation time and supplier protocol. pAAV-Ef1a-DIO hChR2(C128S/D156A)-EYFP was a gift from Karl Deisseroth (Addgene plasmid # 35503). FCK-ChrimsonR-GFP was a gift from Edward Boyden (Addgene plasmid # 59049). Third-generation lentiviruses were produced by transient four-plasmid co-transfection into HEK293T cells using the calcium phosphate transfection method. Supernatants were collected, passed through a 0.45 μm filter and purified by ultracentrifugation.

### Electrophysiology and Light Stimulation Protocols

Whole-cell patch-clamp recordings of cultured neurons were performed at room temperature using patch pipettes (4–8 MΩ), after attaining GΩ patch seals. Traces were acquired in current-clamp mode using HEKA EPC10 amplifier and digitizer, and Patchmaster software (HEKA). The extracellular solution contained NaCl (135 mM), KCl (5.4 mM), MgCl_2_ (1 mM), CaCl_2_ (1.8 mM), HEPES (5 mM), and glucose (10 mM), and was adjusted to pH 7.4 with NaOH. The intracellular solution contained K-gluconate (126 mM), KCl (4 mM), MgSO_4_ (1 mM), CaCl_2_ (0.02 mM), BAPTA (0.1 mM), glucose (15 mM), HEPES (5 mM), ATP (3 mM) and GTP (0.1 mM), and was adjusted to pH 7.3 with KOH. Responses were amplified, low-pass-filtered at 3.9 kHz, digitized at 20–50 kHz, stored, and analyzed with Matlab (Mathworks). For photoactivation of young neurons expressing SSFO, 500 ms pulses were applied with either full duty cycle continuous DC bias, or by 50% duty cycle stimulus with sub pulse frequencies of 10 kHz, 5 kHz, 50 Hz. For experiments on mature neurons expressing SSFO, 2,000 ms pulses driven with 10 kHz sub-pulses at a 50% duty cycle were employed. Experiments on SO-PPV based device for excitation of ChrimsonR-expressing culture employed 50 ms and 2,000 ms stimuli, composed of 10 kHz sub-pulses at 50% duty cycle. Unless otherwise stated, a 30 V bias was employed to drive the organic semiconductor based diodes.

### Safety

No unexpected significant hazards or risks were associated with the reported work. All animal manipulations and procedures were performed in accordance with the guidelines established by the European Community Council (Directive 2012/63/EU of 22 September 2010) and were approved by the Italian Ministry of Health.

### Statistical Analysis

Statistical tests were selected based on the data distribution. Normal distribution of the data was verified using the D'Agostino and Pearson omnibus normality test (*p*-value = 0.05). The sample size was indicated as number of recorded neurons (n). Analysis was carried out with Graphpad Prism 5.01 (Graphpad Software Inc.).

## Results

### Fabrication and Biocompatibility of OLEDs Used for Optogenetic Activation

**Figure 1:**
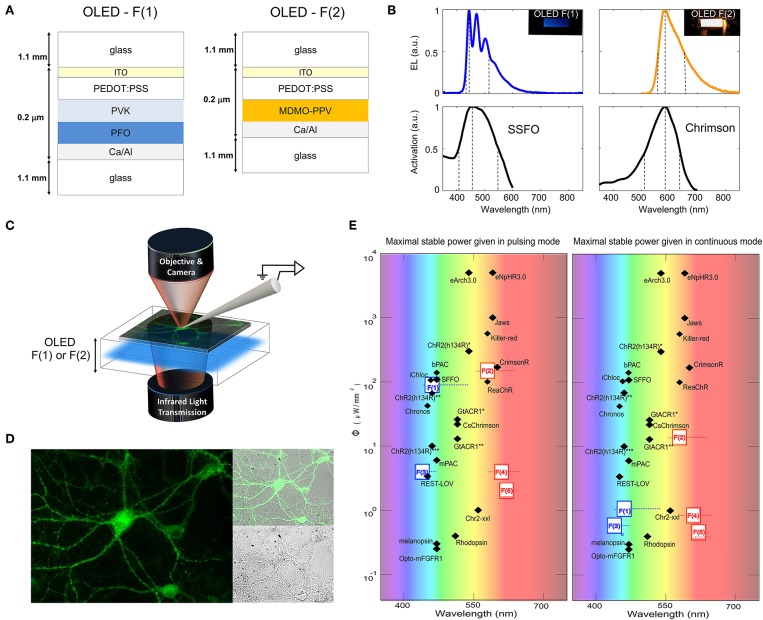
Blue and orange OLEDs based on PFO and SO-PPV use for photo-activation of SSFO and ChrimsonR, respectively. Device architectures **(A)** and emission spectra **(B)** for blue PFO and orange SO-PPV OLEDs. Due to the use of a thin (<100 nm) Al-capping layer, both sets of devices were translucent in the near infrared range, allowing simultaneous photo-stimulation and patch-clamp recordings guided by infrared transmission and epifluorescence. **(C)** Due to the use of a thin (<100 nm) Al-capping layer, both sets of devices were translucent in the near infrared range, allowing simultaneous photo-stimulation and patch-clamp recordings guided by infrared transmission and epifluorescence. **(D)** Fluorescence images of neurons expressing eGFP and located at the surface of the blue PFO OLED, alongside the near infrared transmission image. **(E)** Maximal optical output of solution-processed blue and red-shifted OLEDs with respect to the activation requirements of a toolbox of optogenetic probes. Fluorescent OLEDs are represented with their active layer number F(x) and following Glass/ITO/Pedot:Pss/F(x)/Ca/Al structure with PFO and PVK for F(1) devices; Super Orange (SO-PPV) for F(2) devices; PFO for F(3); MEH-PPV for F(4); CN-PPV for F(5). References for OPSIN tools are: ChR2-XXL (Dawydow et al., 2014); Rhodopsin (Cehajic-Kapetanovic et al., 2015); Jaws (Chuong et al., 2014); ChR2(H134R) (Yu et al., 2015); Killer Red (Williams et al., 2013); GtACR1*, GtACR1** (Govorunova et al., 2015); CsChrimson (Mohammad et al., 2017); Melanopsin (Beiert et al., 2014); bPAC (Stierl et al., 2011); REST-LOV (Paonessa et al., 2016); ChR2(H134R)* (Reinbothe et al., 2014); C1V1(E162T) (Packer et al., 2012); mPAC (Raffelberg et al., 2013); Opto-mFGFR1 (Grusch et al., 2014); ChR2(H134R)** (Maimon et al., 2017); ReaChR1 (Kaufmann et al., 2017); eNpHR3.0, eArch3.0 (Mattis et al., 2011); Chronos (Klapoetke et al., 2014); iChloC (Wietek et al., 2015).

Two optogenetic actuators were investigated for neural control: the blue light-activated, bi-stable SSFO (Yizhar et al., 2011) and the red light-activated ChrimsonR (Klapoetke et al., 2014). Both actuators are light-gated cation channels targeted to the cell membrane, whose activation results in inward (depolarizing) currents when expressed in neuronal cells. Following a screening of candidate light-emitters capable of matching the activation spectra of the two opsins (taking into account emission spectra, achievable emission intensities, and commercial availability of the materials, Figure S1), the blue fluorene-based polymer poly(9,9-di-n-octylfluorenyl-2,7-diyl) (PFO) was selected for SSFO activation, and the orange phenyl alkoxyphenyl based PPV copolymer Livilux PDO-124 (SO-PPV) was selected for ChrimsonR activation (Figures 1A,B). Blue OLEDs were fabricated by sequentially spin-casting onto indium tin oxide (ITO)-coated glass layers of poly(3,4-ethylendioxythiophene : poly(styrene-sulfonate) (PEDOT:PSS) as an anode modifier, poly(9-vinylcarbazole) (PVK) as a hole-transporter and electron-blocker, and PFO as a blue emissive layer (peak emission: 469 nm); orange OLEDs were fabricated by sequentially spin-casting layers of PEDOT:PSS and SO-PPV as an orange emissive layer (peak emission: 573 nm). Both sets of devices were completed by evaporating an aluminum-capped calcium cathode on top of the emissive layer, prior to encapsulation of the exposed surface using UV-curable epoxy resin and a glass cover-slide. The devices were rendered translucent in the near infrared range by restricting the thickness of the Al capping layer to < 100 nm, resulting in a full device transmission of ~5% for the blue OLED and ~4% for the orange OLED at 650 nm and ~8% for the blue OLED and ~7% for the orange OLED at 850 nm (peak). In this way, it was possible to directly image neurons and carry out patch-electrode measurements using a standard patch-clamp set-up equipped with an infrared transmission light path coupled to a CCD camera (Figures 1C–E).

The main challenge is to ensure that OLEDs can operate in a highly saline and biologically active aqueous environment. Finding materials that are inherently stable in the environment in which they function is key to device optimization. We recently demonstrated the photophysical stability of light emitting polymers after extensive immersion to cell-culture medium (Matarèse, 2017). In addition, we have demonstrated the stability and biocompatibility of key OLED electrodes (ITO, gold, aluminum, and silver) when exposed to the physiological milieu (Falco et al., 2016; Matarèse et al., 2018). Nonetheless, the difficult challenge of implantable fully biocompatible OLEDs remain on the electrode injection materials used in devices that are inherently very sensitive to moisture and oxygen (Matarèse, 2017).

In the devices used for this study, the active materials are isolated from the biological medium by a thin glass encapsulant. While the long-term goal is to achieve fully encapsulated and biocompatible devices for implantation, we aimed at demonstrating the suitability of OLEDs for neuronal stimulation to yield the necessary characterization of the encapsulated device for its ability to induce a reliable and high temporal-resolution control of neuronal activity *in vitro*. In view of future developments of flexible devices for bio-implantation, we also preliminarily assessed the cell biocompatibility of the active emissive layer materials that may occasionally get in contact with the biological environment *in vivo*.

When the viability and the basic physiological properties of primary hippocampal neurons on spin-coated polymeric films were measured, no significant differences between neurons grown on the polymers and those grown on glass controls were observed (Figure S2), confirming the biocompatibility of the used materials, similarly to what previously shown for other organic semiconductors (Perylene P13, P3HT; Ghezzi et al., 2011, 2013; Toffanin et al., 2013).

### Performance of OLEDs Under Pulsed Excitation

**Figure 2:**
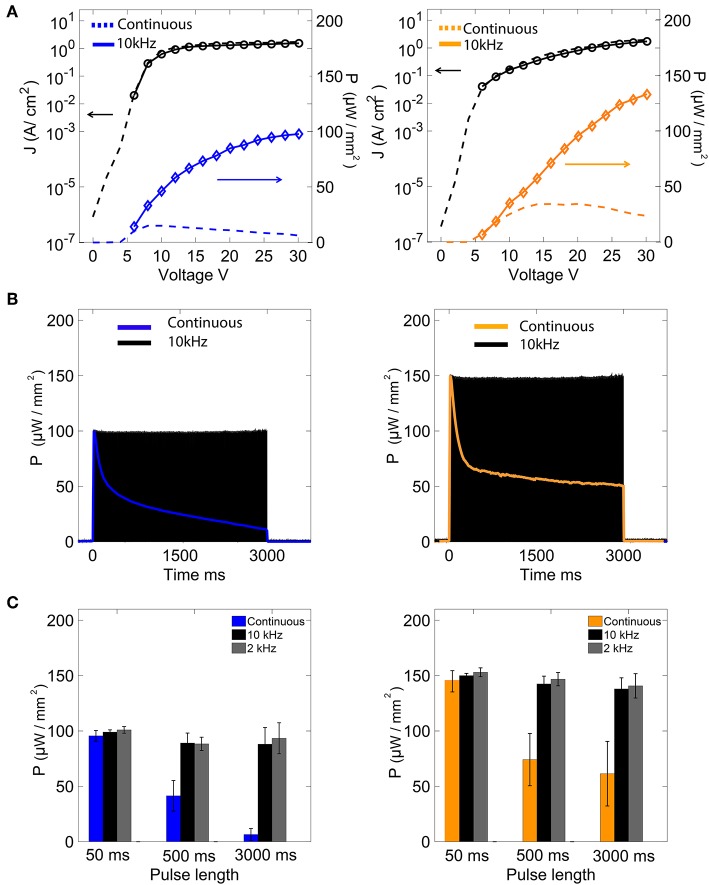
Performance characteristics of blue and orange OLEDs. **(A)** Current-density and optical power vs. voltage characteristics for blue and orange devices under DC and 10 kHz square-wave operation. **(B)** Optical output power vs. time for blue and orange OLEDs under DC and 10 KHz square-wave operation. **(C)** Peak optical power (mean ± sem) under DC and square-wave operation for measurement times of varying length.

Under typical DC operating biases of < 8 V, OLEDs commonly provide optical output power densities ranging from hundreds to tens of thousands of nW/mm^2^ (Lochner et al., 2014; Smith et al., 2014; Morton et al., 2016). However, the typical excitation levels required for optogenetics range from hundreds to thousands of μW/mm^2^ (Mattis et al., 2011; Dawydow et al., 2014; Reinbothe et al., 2014; Mohammad et al., 2017). For instance, under continuous bias operation, our blue OLEDs emitted a peak optical power of 15 μW/mm^2^ at 8 V, while our orange OLEDs emitted a peak optical power of 35 μW/mm^2^ at 15 V (Figure 2A). Increasing the DC bias further resulted in rapid degradation of the devices due to excessive Joule heating, and hence higher optical powers could not be reliably achieved under DC conditions.

Pulsed mode operation—in which devices are driven using low to moderate duty cycle high frequency biases—has previously been shown to improve the efficiency of OLEDs, allowing the use of lower drive voltages and/or current densities for a given optical output power (Nakanotani et al., 2005). This was found to be the case for both our blue and orange OLEDs which, compared to DC operation at the same voltage, showed slightly lower current densities and substantially higher output powers when driven with a 10 kHz square wave in the range 6–30 V (Figure 2A). Pulsed mode operation has the further advantage of improving the operating lifetime of OLEDs with respect to continuous operation: under DC operation at 30 V, the output power of our devices dropped to 50% of the starting value within ~250 ms; under square-wave operation at the same voltage, by contrast, the output power remained approximately constant for over 3 s (retaining ~98% of the starting value; Figure 2B). Moreover, using pulsed operation, devices could still operate upon repetition of the protocol over 30 min. Figure 2C shows the average of each peak optical power over time of the blue and orange OLEDs operated under DC and square-wave operation (two test frequencies of 2 and 10 kHz) over operating times of 50, 500, and 3,000 ms. For operating times < 50 ms, the devices were stable in both DC and square-wave modes, with outputs of about 100 and 150 μ*W*/mm^2^ at 30 V for the blue and orange OLEDs, respectively. However, for longer operating times, a clear difference was observed between DC and square-wave operation. For instance, after 3 s of DC operation, the optical output powers of the blue and orange OLEDs had decreased by approximate factors of ten and two; under 3 s of square-wave operation, by contrast, the optical powers had dropped by <0.9 percent for both OLEDs (Figure 2C).

Low values of external quantum efficiency (EQE) of OLEDs are observed at high current densities (>1 A/cm^2^) for all devices under kHz and DC conditions (Figure S3). It is clear that operation at 10 kHz allows for much longer operation of the OLED compared to DC conditions, but it does not appear to improve massively the quantum efficiency for short pulse lengths. For both OLED devices, the longer was the illumination pulse, the lower the external quantum efficiency was observed (Figure S3 and Table 1).

**Table 1:** Summary of EQEs and temperature rise at different pulse durations for DC and 10 kHz operation modes.

**Operation mode**		**Blue OLED**			**Orange OLED**		
**Pulse period**	**Pulse duration (ms)**	**Optical power (μ*W*/*mm*^2^)^**a**^**	**Temperature rise (°C)^**b**^**	**EQE (%)**	**Optical power (μW/mm^**2**^)^**a**^**	**Temperature rise (°C)^**b**^**	**EQE (%)**
DC	50	96	0.02	0.29	145	0.05	0.38
10 kHz	50	99	0.006	0.34	150	0.03	0.46
DC	500	41	0.27	0.12	74	0.53	0.19
10 kHz	500	89	0.14	0.30	143	0.25	0.43
DC	3,000	6.5	0.74	0.02	61	1.13	0.16
10 kHz	3,000	88	0.40	0.29	138	0.69	0.42

a *Average optical power during pulse duration*.

b *Temperature rise measured at 3,000 ms*.

### Pulsed Operation Reduces the Increase in Surface Temperature

When used *in vivo*, Joule heating within the OLEDs could elevate the local temperature to levels that may become potentially harmful to cells and tissues. Moreover, a temperature increase can induce changes in membrane capacitance and conductance that would depolarize neurons and thus confound the effects of OLED-stimulated opsins. The maximum temperature induced by an LED is going to depend not only on the power dissipated in the LED, but also the heat capacity of the apparatus and how it is in thermal contact with its surroundings. Here, we only focused on surface temperature as control measurement of sub-millisecond single pulse for this organic LED-optogenetics platform.

**Figure 3:**
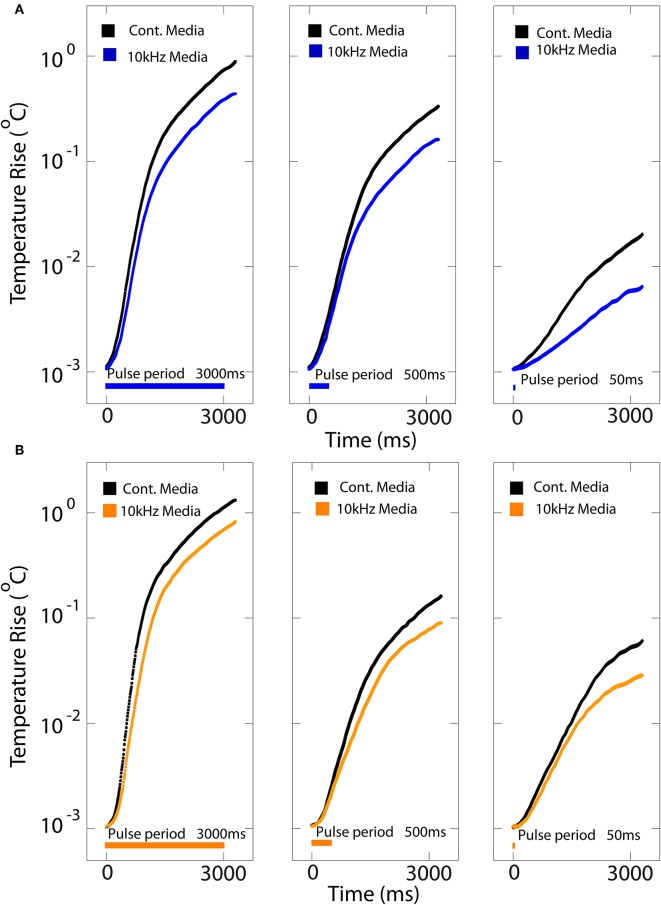
Heating profile of blue and orange OLEDs. The steady-state temperature rise at the surface of a coverslip positioned onto the blue **(A)** and orange **(B)** OLEDs immersed in cell-culture medium vs. time for either DC (cont.; black traces) and 10 kHz square-wave (colored traces) stimulation at 30 V optical power corresponding to for blue and for orange OLEDs for pulse durations of 50, 500, and 3,000 ms.

We monitored temperature as control measurement for the following *in-vitro* experiments using a thermocouple positioned on the anode side of the organic LED on top of the culture coverslip immersed in either ambient air or cell medium for various pulse lengths (Figure 3 and Figure S3). We measured the surface temperature for stimuli ranging from 50 to 3,000 ms, mimicking the light stimulation performed with neurons expressing either SSFO or ChrimsonR (Figures 3A,B). The steady-state temperature rise vs. time for three increasing pulse durations (50, 500, and 3,000 ms) show insight on the maximum average power that can be safely delivered using this platform for a given DC and 10 kHz OLED operation. As expected, the steady-state temperature increases as a function of the pulse duration for both continuous DC and 10 kHz operation modes. With an average power of ~150 μW/mm^2^ for orange OLEDs and ~100 μW/mm^2^ for blue OLEDs, they can safely operate with the KHz mode with temperature increases <1°C (measured at the end of the pulse; <2°C measured at 7 s) for the maximum stimulation period of 3,000 ms while, for the very short pulse period of 50 ms, the temperature increase is only < 0.1°C both with DC and KHz modes (Figure 3 and Figure S3).

For both DC and pulsed operation in cell medium, the temperature change was strongly dependent on the operating bias. For a DC bias of 7 V, the increase in surface temperature after 7 s (with OLEDs operated for the first 3 s) was a few tenths of a degree for both the blue and orange devices, while it was ≈ 2°C for the blue device and ≈3°C for the orange device for a DC bias of 30 V (Figures S3a,b,d,e for the blue and orange OLEDs, respectively). Under 10 kHz square-wave operation, the temperature changes were approximately half the size of the DC case (e.g., ≈ 1–1.5°C at 30 V), consistent with the 50% duty cycle of the square-wave drive scheme. As expected, the temperature increased with the OLED operating time, with broadly similar temperature rises observed when the devices were in contact with cell culture medium or with air (Figures S3b,e). Notably, these temperature changes did not elicit any depolarization in control neurons not expressing opsins (see below).

The analysis of the external quantum efficiency (EQE) for the blue and orange OLEDs for increasing pulse durations (50, 500, and 3,000 ms) revealed that EQE was relatively similar between continuous and pulsed (10 KHz) operation at the shortest pulse duration (50 ms). However, at longer times, EQE sharply dropped with DC operation, while it remained relatively constant for pulsed operation (Figures S3c,f and Table 1).

### Depolarization of SSFO-Expressing Cortical Neurons by Blue OLEDs

**Figure 4:**
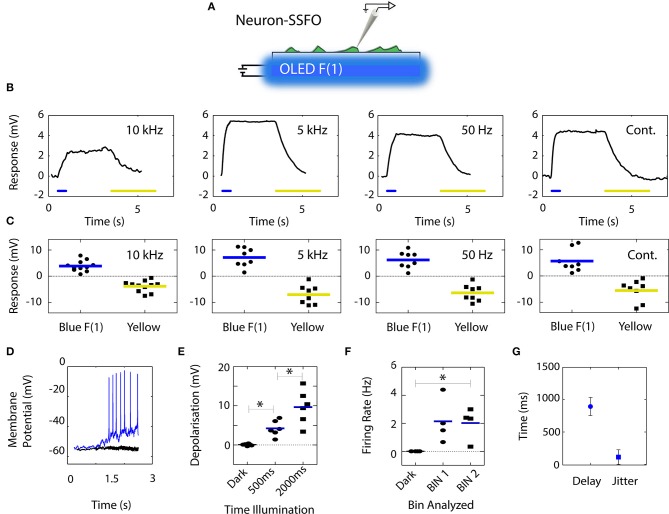
Depolarization of SSFO-expressing neurons by blue OLED illumination at various pulse rates. **(A)** Neurons were transfected with SSFO, stimulated by a blue OLED, and recorded in current-clamp configuration. **(B)** Representative responses from individual neurons. At all tested frequencies, 500 ms of blue light stimulation was sufficient to depolarize the neurons, leading to sustained activation that extended beyond the pulse stimulus; activation could be fully reversed by yellow light stimulation. **(C)** Individual neurons (symbols) and mean (colored lines) amplitudes of blue light-induced depolarization in response to 500 ms stimuli and yellow light-induced hyperpolarization in SSFO-expressing neurons (*n* = 8–11). **(D)** Sample recording from a mature neuron transfected with SSFO (blue trace) and illuminated for 2 s that successfully reached the firing threshold. The black trace represents the response of a control mock-transfected neuron. **(E)** Effects of stimulus length on the extent of depolarization, excluding action potentials, of SSFO-expressing neurons in response to 2 s stimuli (symbols and lines represent individual neurons and the mean, respectively). Depolarization increased significantly with the time of illumination (*n* = 6). **(F)** The firing rate of SSFO-expressing neurons increased significantly during prolonged illumination. The individual values (symbols) and mean (line) firing are shown for 1 s bins before (dark) and during the blue light stimulus (*n* = 4). **(G)** Quantification of the delay and jitter of action potential firing based on the first spike per stimulus calculated across all recorded neurons.

The coupling of the blue OLED emission to opsin-expressing neurons was investigated using a low-density culture of cortical neurons prepared from E18 mouse embryos. Primary neurons were transfected at 6–8 days *in vitro* (DIV) with the bi-stable opsin SSFO (Yizhar et al., 2011; Figure 4A); measurements were carried out 24 h after transfection. SSFO has one of the lowest recorded Effective Power Densities for 50% activation (EPD_50_; Mattis et al., 2011), lying between 0.1 and 10 μW/mm^2^, significantly lower than the measured output of our blue OLEDs in square-wave operation. The low activation power is due to the bi-stable nature of the SSFO opsin channel, which remains in an open conductive state after illumination with blue light, closing with a long relaxation time of 29 min (Yizhar et al., 2011). This means sequential pulses of light have a cumulative effect on the open channel populations, bypassing the current decay due to the rapid channel inactivation typically experienced by other opsins. Channel closure can be hastened by a secondary pulse of yellow light, causing the membrane potential to return to its resting value (Yizhar et al., 2011). Cell membrane depolarization under blue-light illumination followed by repolarization under yellow-light illumination consequently provides a clear indication that the initial depolarization is attributable to SSFO.

Firstly, we compared the effects of 500 ms stimuli, driven by a DC bias and 10 kHz, 5 kHz, and 50 Hz square waves. SSFO-transfected neurons plated on glass coverslips were positioned at the surface of the OLED's anode-side surface (75 mm^2^ area), with the OLED surface and coverslip bathed in extracellular solution for patch-clamp recordings (Figure 4A; see also Figure 1C). Changes in membrane potential induced by the various light stimulation protocols were recorded in current-clamp mode, with each stimulus being repeated five times per recorded cell. The representative traces reported in Figure 4B show that in all cases the emission from the blue OLED successfully activated the SSFO channels and had a depolarizing effect on the recorded neurons. At all tested stimulation frequencies, the depolarization elicited by the blue OLED extended well beyond the 500 ms duration of the blue light illumination window, and was readily reversed when the neuron was exposed to yellow light (derived from an external yellow inorganic LED focused through the microscope objective), confirming that the voltage responses recorded in the neurons were indeed driven by SSFO-generated currents (Yizhar et al., 2011) (Figure 4B). The mean (±sem) response amplitudes measured in the analyzed neurons did not vary significantly across the tested sub-pulse rates (depolarization: 3.9 ± 0.6 mV, *n* = 11; 7.2 ± 1.3 mV, *n* = 8; 6.2 ± 1.1 mV, *n* = 8; and 5.7 ± 1.5 mV, *n* = 8; for 10 kHz, 5 kHz, 50 Hz, and continuous operation, respectively one-way ANOVA; *p* > 0.05) (Figure 4C).

We next used a prolonged 2-s stimulation time to drive the SSFO-expressing neurons to the firing threshold. A drive frequency of 10 kHz was employed to ensure sustained device performance and a minimal temperature increase (see Figures 2, 3). Most of the recorded SSFO-expressing neurons (*n* = 4 out of 6 neurons) fired multiple action potentials during the period of light emission (Figures 4D–F). A significant increase of firing rate was measured during the illumination period (Kruskal-Wallis/Dunn's tests, *p* < 0.05; vs. baseline, *p* < 0.05). The first spike elicited per stimulus occurred with a delay of 893.7 ± 139.9 ms relative to the light stimulus onset and a jitter of 139.9 ± 112.4 ms (calculated as the standard deviation of the latency) (Figure 4G). The remaining cells (*n* = 2 out of 6) exhibited a substantial depolarization (6.84 ± 2.12 mV over the 2 s illumination period), but did not reach the firing threshold. Mock-transfected neurons, subjected to the same light stimulation conditions, showed no depolarization (−0.57 ± 1.08 mV) and no firing during/after the light stimulus.

### Millisecond-Scale Control of Spike Firing by Orange OLEDs in ChrimsonR-Expressing Neurons

**Figure 5:**
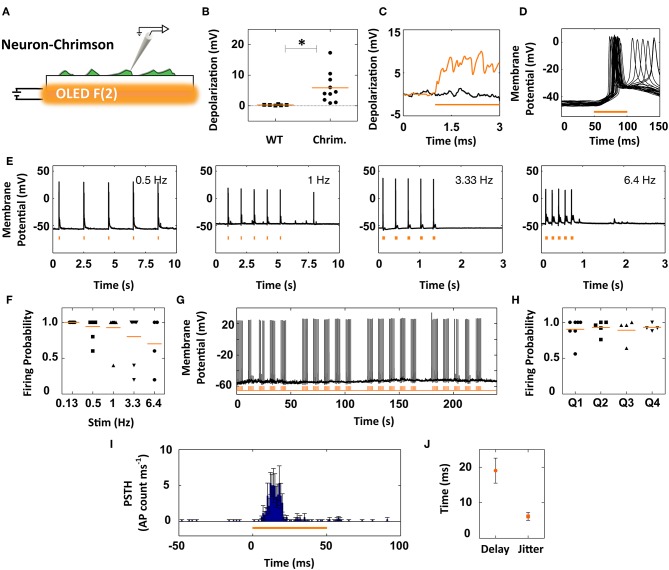
Coupling of orange OLEDs to ChrimsonR-expressing neurons for millisecond control of action potential firing. **(A)** Primary neurons were transduced with lentiviral vectors encoding ChrimsonR, and responses to orange OLED illumination were recorded by patch-clamp. **(B)** Subthreshold responses to 50 ms illumination in ChrimsonR-expressing cells. Control mock-transduced neurons (WT) showed no responses to light (*p* < 0.05, Mann-Whitney *U*-test). **(C)** Representative subthreshold responses to prolonged (2 s) illumination of ChrimsonR-expressing cells (orange trace) and mock-infected neurons (black trace). **(D)** Overlay of membrane responses of a single ChrimsonR-expressing neuron to 25 repetitions of 50 ms pulses, successfully triggering action potential firing. **(E)** Representative traces of neurons successfully firing action potentials during five consecutive 50 ms light stimuli at various inter-stimulus repetition rates. **(F)** Firing probability vs. stimulus frequency (0.13–6.4 Hz), calculated over first five stimuli per cell (symbols); orange lines represent the group means. **(G)** Representative spiking activity in response to five trains (each composed of five 1 Hz-stimuli) administered at 0.1 Hz. **(H)** Firing probability over multiple repetitions (25–100) following the stimulation protocol shown in **(G)** for individual cells (symbols) and group means (lines). **(I)** Peristimulus time histogram for the time window 50-ms-before/50-ms-after the stimulus (1 ms bins). **(J)** Quantification of the delay and jitter of action potential firing based on the first spike per stimulus calculated across all recorded neurons.

The multi-second stimulation procedure used to activate SSFO by the blue OLEDs is suitable for slow tasks like photo-mediated insulin release or photo-regulation of gene expression, where control on the second-to-minute timescale is typically sufficient. However, many optogenetic applications require opsin currents to be switched on and off with sub-second temporal precision. In addition, it is often preferable to use red light for opsin stimulation since red light penetrates more deeply into tissue and results in lower phototoxicity. We therefore assessed the feasibility of coupling our orange OLEDs to the rapidly inactivating, red shifted opsin ChrimsonR (Chaffiol et al., 2017). To gain high and widespread opsin expression levels in the primary neuronal networks, ChrimsonR was packaged in a lentiviral vector with a high efficiency of neuron transduction (Figure 5A).

ChrimsonR-expressing neurons displayed both sub-threshold depolarizations and light-mediated action potentials in response to stimulation by the orange OLED (50 ms, 10 kHz, 30 V), while non-transduced neurons were unresponsive to the same light stimuli (Figure 5B and Figure S3a). ChrimsonR-expressing neurons displayed sub-threshold activations of 5.89 ± 1.52 mV (mean ± sem; *n* = 10) during the short 50 ms stimuli (Figure 5B). These sub-threshold depolarizations were reliably elicited across multiple OLED light stimulations (Figure S3b). In contrast to the SSFO-expressing neurons excited by our blue OLEDs, prolonged illumination (2 s, 10 kHz, 30 V) of ChrimsonR-expressing neurons by the orange OLEDs led to a mean (±sem) depolarization of 4.3 ± 1.5 mV (*n* = 3) during the first 500 ms of illumination that did not further increase during the remainder of the light pulse (4.01 ± 1.6 mV at 2,000 ms; see sample trace in Figure 5C).

The largest subset (*n* = 13 out of 26 neurons) of ChrimsonR-expressing neurons showed a reproducible firing pattern in response to light, successfully reaching the firing threshold within the 50 ms stimulus time (Figure 5D). To check the temporal precision of neuronal activation by the OLED-opsin system, we delivered consecutive light stimuli (10 kHz, 50 ms) at various modulation frequencies (0.5–6.4 Hz). As highlighted by the representative traces of Figure 5E, action potential firing faithfully followed the light cues. The ChrimsonR/orange-OLED system achieved a 100% firing probability at 0.13 Hz (*n* = 6), 94% at 0.5 Hz (*n* = 10), 92.5% at 1 Hz (*n* = 8), 80% at 3.3 Hz (*n* = 7), and 70% at 6.4 Hz (*n* = 4) showing that, except for occasional failures, neuronal firing patterns were tightly locked to the optical output of the OLED (Figure 5F).

To test the reliability of the system, the neurons were exposed to a long series of repeated stimulations (25–100 stimuli), while holding them under patch-clamp conditions. The experimental protocol employed trains of stimuli composed of five bursts (0.1 Hz bursting rate) containing five 50 ms stimuli administered at 1 Hz (10 kHz square wave). The trains were administered up to four times per cell (Figure 5G). The mean firing probability per cell was quantified for each train (5 packets of 5 stimuli), revealing a reliable elicitation of action potentials (mean quartile probability values: Q1 = 90.5%, *n* = 7; Q2 = 93% *n* = 5; Q2 = 89%, *n* = 4; Q4 = 93%, *n* = 4) (Figure 5H). A similar performance was obtained using 2-s interstimulus intervals (Q1 = 94%, *n* = 4; Q2 = 91% *n* = 3; Q2 = 79%, *n* = 3; Q4 = 76%, *n* = 3). The peristimulus time histogram (PSTH) calculated from the cumulative action potential distribution per cell (Figure 5I) revealed that the increased firing probability closely followed the onset of the light stimulus. Taking into account all firing neurons tested with the orange OLEDs, the mean (±sem) time between light onset and the first action potential per stimulus was determined to be 19.2 ± 3.3 ms, with a jitter of 6.2 ± 1.1 ms (Figure 5J). In contrast to ChrimsonR-expressing neurons, non-transduced cells present in the same neural networks were virtually unresponsive to light (mean depolarization in light = 0.09 ± 0.32 mV, *n* = 6), except for rare and delayed responses likely mediated by synaptic network connectivity to opsin-expressing neurons (Figures S3c,d). In summary, the responses documented here highlight the ability of the orange OLED/ChrimsonR couple to photo-stimulate neurons, showing that light-emitting diodes based on organic conjugated polymers can straightforwardly be used to elicit neuronal activation.

## Discussion

The above results indicate the suitability of OLEDs for the optogenetic activation of primary neurons. Devices based on PVK/PFO and SO-PPV were fabricated and characterized and their performance under continuous DC bias and pulse mode operation was compared. While under DC operation, the devices degraded rapidly within a few hundred milliseconds (below the optical powers required for optogenetic stimulation), using square-wave frequencies of 5 kHz and above, they exhibited stable and high brightness output of >100 μW/mm^2^ when operated at 30 V. For the first time kHz modulation has been applied to OLEDs for optogenetics applications. Two OLED-Opsin pairs were tested for time-locked neuronal activation with multi-kHz pulsing mode. Blue OLEDs successfully stimulated neurons expressing SSFO by inducing a sustained depolarization that persisted beyond the illumination period, and was readily switched off when the neuron was exposed to yellow light, as expected for SSFO-generated currents (Yizhar et al., 2011). Orange OLEDs effectively stimulated neurons expressing the fast inactivating ChrimsonR opsin by eliciting firing patterns were tightly time-locked to the OLED emission that are suitable for most optogenetic applications where opsin currents must be switched on/off on a sub-second timescale.

Previous results using non-solution processed OLEDs, characterized by complex structures and small molecule-type of devices were only able to activate inward currents in opsin-expressing cell lines or induce behavioral changes in opsin-expressing algae or Drosophila larvae on a second scale (Steude et al., 2015, 2016; Morton et al., 2016). Instead, our polymeric light emitting devices made of solution-processable materials were capable to achieve not only of fluctuations in the membrane potential, but a real millisecond-scale control of neuronal firing that is essential for any neuroscience application.

Inorganic LED solutions offer high optical power. However, they lack precise matching with with the opsin activation spectra. Inorganic LEDs are a chip point source (small pixel area) with high power density produced. The generated heat, due to the high power, has very little surface area to dissipate. Inorganic LEDs, which produce a large amount of heat during operation, require additional heat sinking for keeping low temperature of devices. One of the attractive features of polymeric OLEDs is the large-area of homogeneous light emission at relatively low temperature. In addition, OLEDs have the advantage to be used for *in vitro* electrophysiology coupled to optogenetics. The OLED-Optogenetic platform proposed here can be used to test OLED prototypes before *in vivo* use on flexible substrates and toward next generation of organic LEDs for optogenetics. The limitations of OLED remain with the relatively short lifetime due to air/aqueous instability of the electron injection layer, which is still a challenge for *in vivo* use with flexible substrates. Our devices could still operate over 30 min, although some degradation of the optical power occurs overtime, suggesting that the device structure should be optimized for allowing lifetimes needed long-term *in vivo* applications. However, there is a high demand to incorporate such OLEDs into implantable devices using flexible substrates such as Silicon, Parylene C, Polymide, or SU8 with appropriate oxygen barrier materials. Although fully polymeric OLEDs are less efficient that inorganic LEDs, the device performance could be further enhanced when using highly sensitive opsins that require lower density operation. This will pave the way for a near future of fully biocompatible, stable and highly performance of fully Polymeric OLEDs.

## Conclusions

Currently encapsulated in glass, the described OLEDs are can be employed as smart culture substrates for *in vitro* optogenetics experiments. Indeed, a series of OLEDs capable of meeting the lowest activation requirements of the most commonly used optogenetic actuators is shown in Figure S4. Future work will focus on the development of more efficient and stable devices that allow using lower drive voltages and extended operation times, as well as on the development of thin-film embeddable devices for *in vivo* optogenetic applications. Ultimately, the promise of OLED technology is a system that is easily implementable, implantable, and devoid of external optics. The rapid pace of progress in OLED and optogenetic technologies will ensure that OLEDs continue to find numerous applications in bioelectronics.

## Data Availability Statement

The authors declare that all data that support the figures within this paper and other findings of this study are hosted at the Istituto Italiano di Tecnologia and can be accessed by contacting the corresponding author.

## Ethics Statement

The animal study was reviewed and approved by Italian Ministry of Health.

## Author Contributions

BM designed, fabricated and characterized devices, and prepared hardware communication protocols for OLED integration with patch-clamp set-up. PF prepared neuronal cultures, produced viruses, carried out electrophysiological recordings, and contributed to device design. JM and FB planned experiments and contributed critically to data analysis and interpretation. All authors contributed to the writing of the manuscript.

### Conflict of Interest

The authors declare that the research was conducted in the absence of any commercial or financial relationships that could be construed as a potential conflict of interest.

## Abbreviations

CN-PPV: poly(2,5-di(hexyloxy)cyanoterephthalylidene)
DI: double distilled
DIV: days *in vitro*
HEK293T: Human Embryonic Kidney-293T
EPD50: effective power density for 50% activation
IPA: isopropanol
ITO: indium tin oxide
MEH-PPV: poly[2-methoxy-5-(2-ethylhexyloxy)-1,4-phenylenevinylene]
OLED: organic light-emitting diodes
PEDOT:PSS: poly(3,4-ethylendioxythiophene:poly(styrene-sulfonate)
PFO: poly(9,9-di-n-octylfluorenyl-2,7-diyl)
PVK: poly(9-vinylcarbazole)
SO-PPV: phenyl alkoxyphenyl-based PPV copolymer Livilux PDO-124
SSFO: sustained step function opsin.
