# Supplementary material for: Sub-millisecond Control of Neuronal Firing by Organic Light-Emitting Diodes
Source: Front Bioeng Biotechnol. 2019 Oct 22;7:278. doi: 10.3389/fbioe.2019.00278 (PMC6817475; doi:10.3389/fbioe.2019.00278)
Supplement: Supplementary file 1 [file Image_1.pdf]

SUB-MILLISECOND CONTROL OF NEURONAL FIRING BY ORGANIC LIGHT-EMITTING DIODES

Bruno F.E. Matarèse[1*°], Paul L.C. Feyen[2,3*°], John de Mello[1,4]†, Fabio Benfenati[2,5]†

[1] Department of Chemistry, Imperial College London, South Kensington Campus, London SW7 2AZ, UK; [2] Center for Synaptic Neuroscience and Technology, Istituto Italiano di Tecnologia, Largo Rosanna Benzi 10, 16132, Genoa, Italy; [3] Department of Experimental Medicine, Section of Physiology, University of Genova, Viale Benedetto XV 3, 16132 Genoa, Italy; [4] Centre for Organic Electronic Materials, Department of Chemistry, NTNU, N-7491 Trondheim, Norway; [5] IRCCS Ospedale Policlinico San Martino, Genova, Italy

*equal contribution
†corresponding authors

SUPPLEMENTARY FIGURES

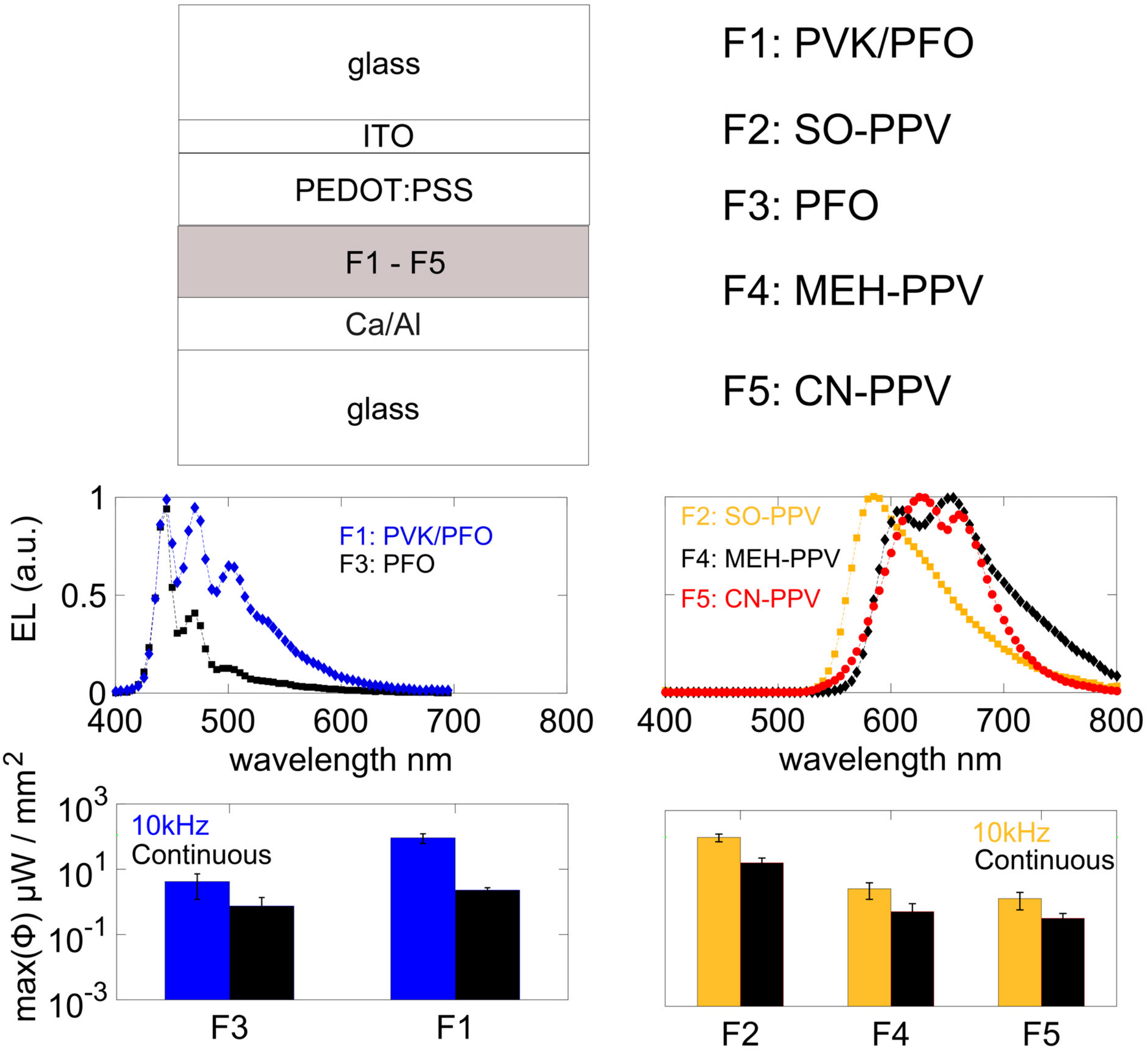

Figure S1. Characterization of a series of blue and red solution-processed OLEDs for optogenetics. *Top:* Device structure and naming representing the device number to their active layer for the fluorescent device structure. *Middle:* Electroluminescent spectra of devices. *Bottom:* Maximal stable output in continuous bias operation (black bars) or pulsed mode operation (10 kHz 50% duty cycle) (colored bars).

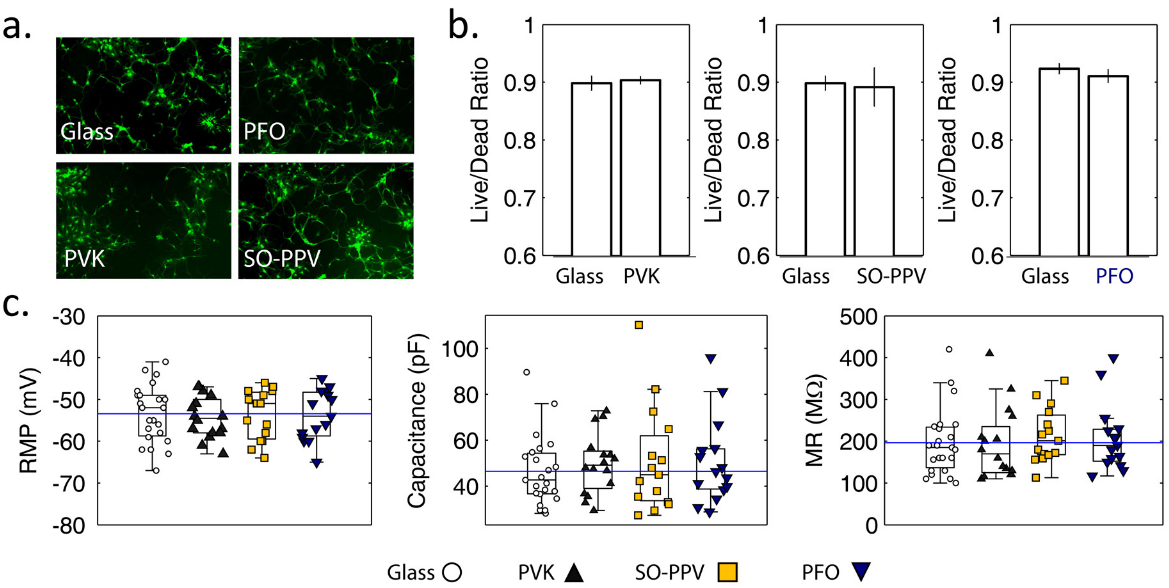

Figure S2. Primary neurons are not affected by conjugated polymers. **(a)** Primary hippocampal neurons prepared from the embryonic brain were plated on semiconducting polymers and recorded by patch-clamp between 14-21 DIV. **(b)** Cell viability was assessed by fluorescein diacetate and propidium iodide staining and expressed as live/dead cell ratio (means±sem). **(c)** Resting membrane potential, capacitance and membrane resistance for the neurons grown over glass *vs* those grown on the tested polymers ($p>0.05$). Individual values and means are shown with symbols and lines, respectively.

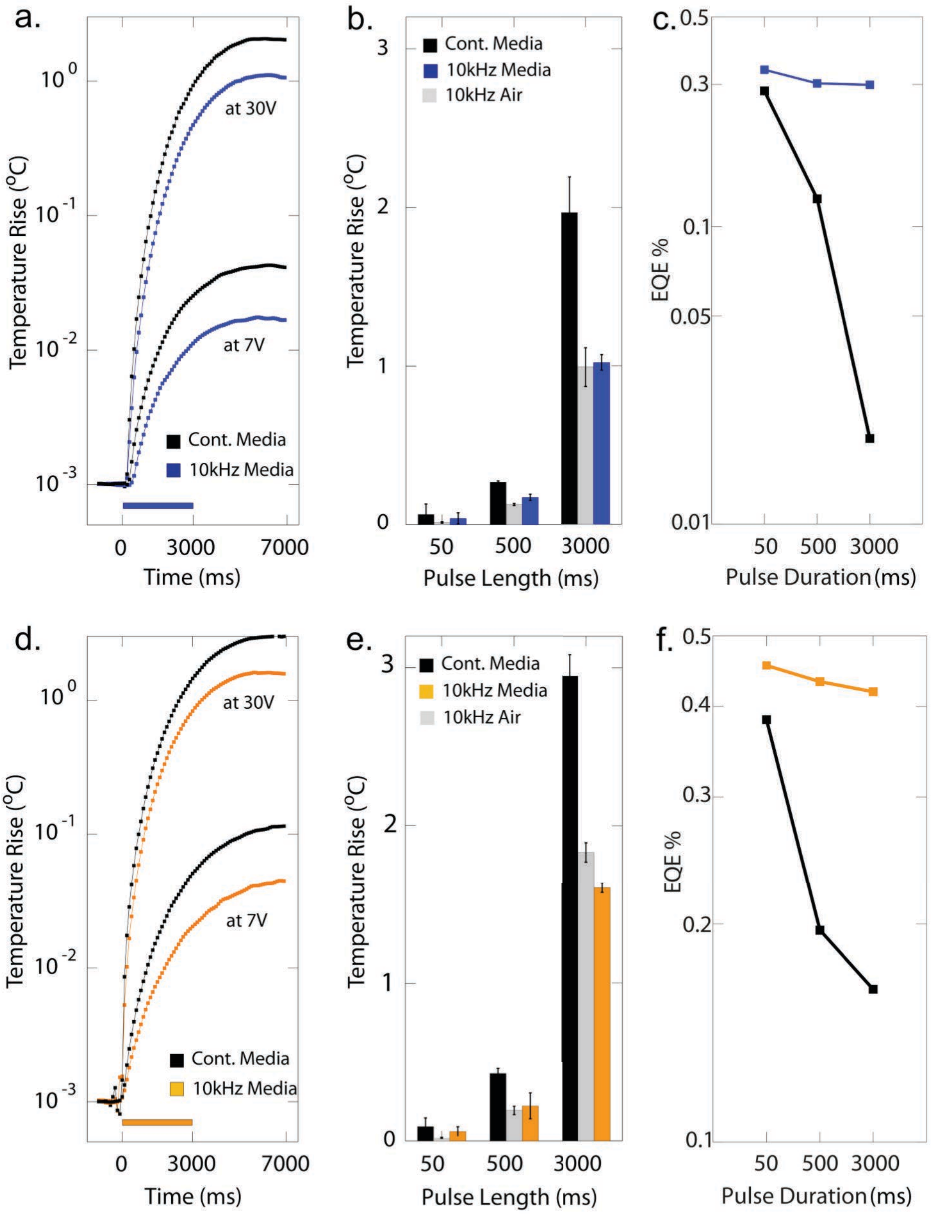

Figure S3. Heating characteristics of blue and orange OLEDs. (a,d) Temperature rise at the surface of a coverslip positioned onto the blue (**a**) and orange (**d**) OLEDs immersed in cell-culture medium *versus* time for either DC (cont; black traces) or 10 kHz square-wave (colored traces) stimulation at either 7 V (lower traces) or 30V (upper traces) drive voltage. The temperature rise was followed for 7 s, with the OLEDs operated for the first 3 s. **(b,e)** Pulse-length dependence of temperature rise under DC and 10 kHz square-wave operation with 30 V drive voltage for blue (**b**) and orange (**e**) OLEDs under the various experimental conditions (cell medium for continuous operation; air and cell medium for pulsed operation). **(c,f)** The external quantum efficiency (EQE) of OLEDs for pulse durations of 50, 500 and 3000 ms for blue (**c**) and orange (**f**) OLEDs.

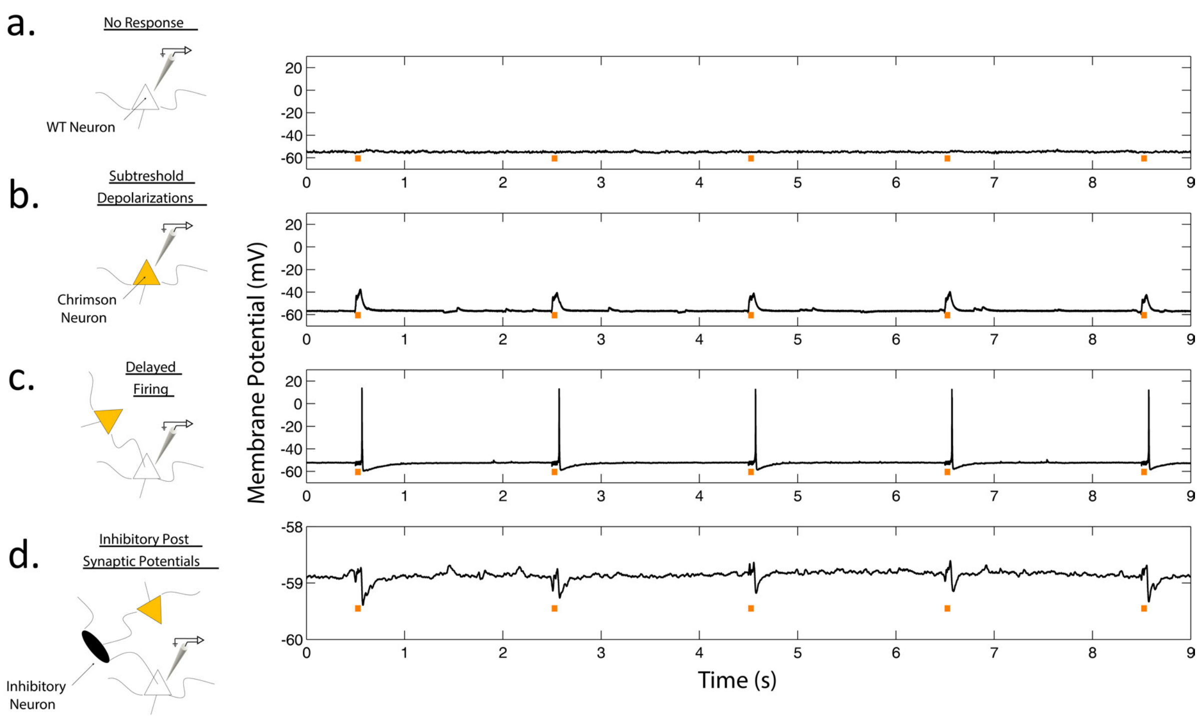

Figure S4. Responses of primary cortical neurons to OLED(F2)-generated light stimuli. ChrimsonR-transduced and mock-transduced (WT) cortical neurons grown under high density conditions were subjected to 50 ms OLED (F2) pulsed light stimuli. **(a)** Representative recording from a WT neuron. **(b)** A ChrimsonR-transduced neuron responds to the optical stimulation with subthreshold depolarizations. **(c)** WT neuron with consistent delayed action potential firing in response to 50 ms illumination. **(d)** Several neurons, including both control and ChrimsonR-expressing cells, displayed inhibitory postsynaptic potentials in response to light.
